# DRAW+SneakPeek: Analysis workflow and quality metric management for DNA-seq experiments

**DOI:** 10.1093/bioinformatics/btt422

**Published:** 2013-08-13

**Authors:** Chiao-Feng Lin, Otto Valladares, D. Micah Childress, Egor Klevak, Evan T. Geller, Yih-Chii Hwang, Ellen A. Tsai, Gerard D. Schellenberg, Li-San Wang

**Affiliations:** ^1^Department of Pathology and Laboratory Medicine and ^2^Institute for Biomedical Informatics, University of Pennsylvania Perelman School of Medicine, Philadelphia, PA 19104, USA, ^3^Department of Physics, University of Washington, Seattle, WA 98105, USA, ^4^Genomics and Computational Biology Graduate Group, University of Pennsylvania Perelman School of Medicine, Philadelphia, PA 19104, USA and ^5^Department of Pathology and Laboratory Medicine, The Children's Hospital of Philadelphia, Philadelphia, PA 19104, USA

## Abstract

**Summary:** We report our new DRAW+SneakPeek software for DNA-seq analysis. DNA resequencing analysis workflow (DRAW) automates the workflow of processing raw sequence reads including quality control, read alignment and variant calling on high-performance computing facilities such as Amazon elastic compute cloud. SneakPeek provides an effective interface for reviewing dozens of quality metrics reported by DRAW, so users can assess the quality of data and diagnose problems in their sequencing procedures. Both DRAW and SneakPeek are freely available under the MIT license, and are available as Amazon machine images to be used directly on Amazon cloud with minimal installation.

**Availability:** DRAW+SneakPeek is released under the MIT license and is available for academic and nonprofit use for free. The information about source code, Amazon machine images and instructions on how to install and run DRAW+SneakPeek locally and on Amazon elastic compute cloud is available at the National Institute on Aging Genetics of Alzheimer’s Disease Data Storage Site (http://www.niagads.org/) and Wang lab Web site (http://wanglab.pcbi.upenn.edu/).

**Contact:** gerardsc@mail.med.upenn.edu or lswang@mail.med.upenn.edu

## 1 INTRODUCTION

Next-generation sequencing (NGS) has redefined what big data means in biomedical research. Advances in quality and capacity have led to a declining cost of implementation, allowing NGS to be used in a wide range of experiments at a variety of scales; from a few samples in small laboratories to thousands of samples from multi-institute collaborations. Processing terabytes of data requires a certain level of information technology and bioinformatics expertise, which can be daunting to small laboratories with limited resources. The programs we developed will enable these groups to process DNA-seq data and identify single-nucleotide variants and small insertions and deletions (indels).

**Fig. 1.**
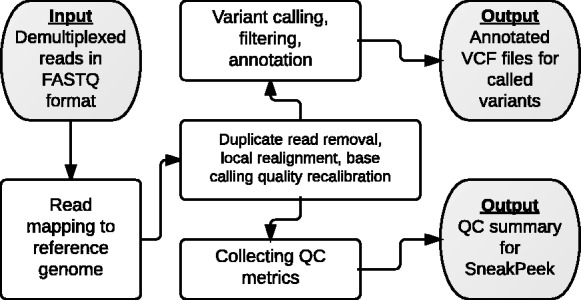
DRAW+SneakPeek overview

Our software suite consists of DNA resequencing analysis workflow (DRAW) and SneakPeek, two independent interoperable components (Fig. 1). DRAW automates the entire workflow of whole-genome/whole-exome DNA-seq data processing from mapping sequence reads to calling variants. SneakPeek is a quality metrics management system for reviewing the sequencing quality of multiple samples across different flow cells. It displays dozens of quality metrics per sample collected throughout DRAW on a dynamic web interface and is a useful diagnostic tool for trouble-shooting unsatisfactory sequencing results.

We used DRAW+SneakPeek to process whole-genome sequencing, whole-exome sequencing (WES) and targeted sequencing experiments on traditional high-performance computing clusters as well as on Amazon elastic compute cloud (EC2). DRAW was used to analyze part of the WES data for a multi-institutional autism study (Neale *et al.*, 2012) and more than 500 exomes/genomes for human and *Caenorhabditis elegans* in our laboratories.

## 2 DRAW: DNA RESEQUENCING ANALYSIS WORKFLOW

We designed DRAW based on the best practice variant detection from Genomic Analysis Toolkit (GATK) (DePristo *et al.*, 2011). DRAW accepts single-end and pair-end reads in FASTQ format from whole-genome sequencing, WES and targeted DNA-Seq experiments. DRAW uses BWA (Li & Durbin, 2009) to map reads to the reference genome, PICARD (http://picard.sourceforge.net/) to mark duplicates and SAMtools (Li *et al.*, 2009) to merge the read alignment Binary version of SAM (BAM) files. GATK performs local realignment near known indel sites, recalibration of base call quality scores, variant calling [Single Nucleotide Polymorphisms (SNPs) and indels], variant filtration and depth and coverage summary. DRAW uses snpEff (Cingolani *et al.*, 2012) to annotate called variants.

To use DRAW, the user first enters sample information into a text file (template and documentation included). DRAW then generates bash scripts for Oracle Grid Engine job submission commands for the user to invoke at the command line. Job dependency and error checking are implemented to streamline and automate the entire workflow. Whenever multi-threading is supported by the third-party programs, jobs will be distributed in parallel computing environment to expedite the process. A complete run of DRAW produces analysis-ready read alignment BAM files, annotated variant call Variant Call Format files and a flat file of 36 quality metrics ready for SneakPeek (Section 3). Should errors occur, the completed steps can be skipped during the re-execution. This modularity reduces potential waste of time and cost on unnecessary repeated jobs.

## 3 SNEAKPEEK: QUALITY METRICS MANAGEMENT SYSTEM

DRAW generates a variety of quality metrics that can be imported into a MySQL database inside SneakPeek. SneakPeak's web interface enables users to quickly assess sequencing quality, e.g. depth coverage on target sites per sample, and identify issues such as excess off-target capture rate. Samples from different flow cells can be placed side by side for comparison to identify problems on the flow cell level. Users can also run SneakPeek without DRAW by supplying their own Quality Control (QC) metrics.

SneakPeek was built on a LAMP (Linux, Apache Httpd, MySQL and PHP) server and the Ext-JS JavaScript web applications framework and requires little storage or computing power. It supports user access control per sequencing project via account/password authentication. Query results can be saved for additional analysis by spreadsheet programs or statistical software such as R.

## 4 USING DRAW+SNEAKPEEK ON AMAZON EC2

We evaluated DRAW on Amazon EC2 using a single flow cell WES dataset with 350.2 billion bases (100 bp pair-end reads from HiSeq 2000 sequencer) from 34 multiplexed samples using Nimblegen SeqCap EZ Human Exome Library. DRAW processed the dataset in 1943.2 core-hours, or 17.4 h/core on 14 quadruple extra large instances (112 cores), saving 1.1 TB data. Total cost was $528 including storage ($95 for 4104 GB disk space over 7 days), computing ($17 for Elastic Block Storage I/O and $270 for CPU time, 52% for read mapping, 38% for base call quality score recalibration and local realignment, 10% for variant calling) and data transfer (upload is free, and downloading 1.1 TB costs $146). A SneakPeek instance on Amazon can be set up using a small instance at $106.30 over 3 years (November 2012 rate). The112-core configuration with 2-day turnaround time offers a good balance between CPU utilization and speed, and is ideal for typical projects run at small research laboratories. If needed, Amazon EC2 allows a user to run more instances than the usual cap of 20 8-extra-large instances, or 640 cores.

We processed another HiSeq 2000 flow cell on both Amazon EC2 and the PGFI cluster at the University of Pennsylvania (1000 cores) and found similar performances (1890 versus 1858 CPU hours). While the $147 saved by running DRAW locally seems substantial with respect to the $528 computing cost, it is minimal, compared with the sequencing cost ($10K∼20K), the expenditure and effort of acquiring your own cluster, and the less quantifiable cost incurred by sharing a local high-performance computing cluster overwhelmed by sequencing experiment projects.

## 5 COMPARISON WITH OTHER TOOLS

While many programs are available for read mapping, variant calling or other aspects of DNA-Seq analysis, only a handful of open-source pipelines that fully incorporate these programs have been published (Table 1). Among them, ngs_backbone (Blanca *et al.*, 2011) provides the most comparable combination of features with DRAW yet it lacks indel calling. Atlas2 Cloud (Evani *et al.*, 2012) provides Amazon EC2 support at a similar cost but does not support read mapping. Crossbow (Langmead *et al.*, 2009) is cloud-enabled but does not explicitly support WES. Both SOAPsnp and SAMtools perform worse than GATK as single-nucleotide variant callers (O'Rawe *et al.*, 2013). SneakPeek offers a comprehensive quality metrics management that is not available in any other open-source DNA-Seq workflows.

**Table 1.** A comparison of DRAW+SneakPeek with other workflows

Workflows	Atlas2 Cloud	Crossbow	Pwrake (Mishima *et al.*, 2011)	NARWHAL (Brouwer *et al.*, 2011)	ngs_ backbone	DRAW+ SneakPeek
Read mapping	—	Bowtie	—	Bowtie/BWA	BWA	BWA
Duplicate marking	—	—	—	—	PICARD	PICARD
Local realignment	—	—	—	—	GATK	GATK
Base quality score recalibration	—	—	—	—	—	GATK
SNP calling	Atlas-SNP2	SOAPsnp	GATK	SAMTools	SAMTools	GATK
Indel calling	Atlas-indel2	—	Dindel	—	—	GATK
Variant annotation	ANNOVAR	—	—	—	(In-house)	SNPEff
WES/Targeted	Yes	—	Yes	—	—	Yes
QC metric management	—	—	—	—	—	SneakPeek
Amazon EC2	Yes	Yes	—	—	—	Yes
